# Mena^INV^ dysregulates cortactin phosphorylation to promote invadopodium maturation

**DOI:** 10.1038/srep36142

**Published:** 2016-11-08

**Authors:** Maxwell D. Weidmann, Chinmay R. Surve, Robert J. Eddy, Xiaoming Chen, Frank B. Gertler, Ved P. Sharma, John S. Condeelis

**Affiliations:** 1 Department of Anatomy and Structural Biology, Albert Einstein College of Medicine, Bronx, NY, USA; 2 Integrated Imaging Program, Albert Einstein College of Medicine, Bronx, NY, USA; 3 Gruss Lipper Biophotonics Center, Albert Einstein College of Medicine, Bronx, NY, USA; 4 Department of Biology and Koch Institute for Integrative Cancer Research, Massachusetts Institute of Technology, Cambridge, MA, USA

## Abstract

Invadopodia, actin-based protrusions of invasive carcinoma cells that focally activate extracellular matrix-degrading proteases, are essential for the migration and intravasation of tumor cells during dissemination from the primary tumor. We have previously shown that cortactin phosphorylation at tyrosine residues, in particular tyrosine 421, promotes actin polymerization at newly-forming invadopodia, promoting their maturation to matrix-degrading structures. However, the mechanism by which cells regulate the cortactin tyrosine phosphorylation-dephosphorylation cycle at invadopodia is unknown. Mena, an actin barbed-end capping protein antagonist, is expressed as various splice-isoforms. The Mena^INV^ isoform is upregulated in migratory and invasive sub-populations of breast carcinoma cells, and is involved in tumor cell intravasation. Here we show that forced Mena^INV^ expression increases invadopodium maturation to a far greater extent than equivalent expression of other Mena isoforms. Mena^INV^ is recruited to invadopodium precursors just after their initial assembly at the plasma membrane, and promotes the phosphorylation of cortactin tyrosine 421 at invadopodia. In addition, we show that cortactin phosphorylation at tyrosine 421 is suppressed by the phosphatase PTP1B, and that PTP1B localization to the invadopodium is reduced by Mena^INV^ expression. We conclude that Mena^INV^ promotes invadopodium maturation by inhibiting normal dephosphorylation of cortactin at tyrosine 421 by the phosphatase PTP1B.

It is estimated that 90% of deaths from solid tumors are not caused by the primary tumor, but the further dissemination and growth of secondary and tertiary tumors at distal sites, known as metastases^1^,^2^. In order to escape the primary tumor, breast carcinoma cells must first penetrate their native basement membrane, the dense extracellular matrix (ECM) of the tumor stroma, and the endothelium basement membrane before entering the bloodstream^3^,^4^. There is increasing evidence that both the basement membrane and dense networks of cross-linked type-1 collagen in the tumor stroma present significant barriers to tumor cell invasion^5^,^6^,^7^. Penetrating these barriers requires invading tumor cells to produce actin-based proteolytic protrusions termed invadopodia^7^,^8^,^9^,^10^.

The level of invadopodium formation and matrix degradation has been shown to directly correlate with invasion both *in vivo* and *in vitro*^10^,^11^,^12^,^13^. Invadopodia have been identified in mammary tumors using *in vivo* imaging^9^,^14^, and inhibition of invadopodium formation reduces invasion, intravasation and metastasis of breast carcinoma cells^9^,^10^,^15^. Recent work has shown that macrophage-induced invadopodium formation is necessary for *in vitro* trans-endothelial migration of breast carcinoma cells derived from patients and breast carcinoma cells entering the bloodstream *in vivo*^15^.

Invadopodium formation and maturation into a proteolytic structure occurs in several stages after stimulation by a growth factor, such as EGF, integrin signaling^16^,^17^ and/or macrophage contact^15^. Within seconds of stimulation, the binding of a complex of proteins to F-actin puncta, including cortactin, cofilin, N-WASP, Nck1, WIP and Arp2/3, forms the invadopodium “precursor” core^13^,^18^,^19^. Approximately 20 s after the formation of this core complex, Tks5 recruitment and binding to phospholipid PI(3,4)P2 begins the process of invadopodium stabilization at the plasma membrane^20^. Further stabilization, protrusion and maturation of the precursor requires cortactin phosphorylation of at least tyrosine 421 by the kinase Arg^17^,^21^,^22^.

Phosphorylation of cortactin at tyrosine 421 recruits the sodium-hydrogen exchanger NHE1, which increases the local intracellular pH and triggers F-actin severing by releasing active cofilin from cortactin^23^,^24^. Cortactin phosphorylation also recruits Nck1/N-WASP to stimulate Arp2/3 activity, which acts synergistically with cofilin severing of actin filaments to promote actin polymerization and invadopodium protrusion^17^,^22^,^25^. The dephosphorylation of cortactin reverses this process thereby inhibiting cofilin activity and actin polymerization^17^,^25^. However, the phosphatases responsible for cortactin dephosphorylation at tyrosine 421 in invadopodia remain unidentified.

Gene expression profiling of invasive and migrating subpopulations of breast carcinoma cells identified an “invasion signature” containing the Arp2/3, cofilin and Mena regulatory pathways that are essential for cell migration and invadopodium formation^26^,^27^,^28^,^29^. Mena is a member of the Enabled/vasodilator-stimulated phosphoprotein (Ena/VASP) family^30^, known to regulate actin cytoskeletal geometry and promote the elongation of actin filaments during membrane protrusion by antagonizing barbed end capping activity^31^,^32^. Mena localizes to the leading edge, focal adhesions and the tips of filopodia via its Ena/VASP Homolog 1 (EVH1) domain^30^,^33^,^34^ and to PI(3,4)P2-rich regions of membrane found in invadopodia^20^,^35^. The EVH1 domain of Mena binds to a consensus motif (FP_4_) on specific targeting proteins, such as Lamellipodin, which localize Mena to each of these locations^20^,^33^,^35^,^36^,^37^. Mena also binds directly to the cytoplasmic tail of the α5 integrin subunit and promotes bi-directional signaling through α5β1 integrin, a major receptor for fibronectin^38^,^39^.

Mena contains several additional exons that may be expressed via alternative splicing to produce isoforms with distinct expression patterns in invasive and non-invasive populations of tumor cells^30^,^40^,^41^,^42^. In particular, the expression of the invasion-associated Mena isoform, termed Mena^INV^, is upregulated in the invasive and migratory tumor cells relative to the non-migratory primary tumor cells^26^,^27^,^28^,^29^,^42^. Expression of the Mena^INV^ isoform, identified by an alternatively-included exon that inserts a 19 amino-acid sequence just downstream of the EVH1 domain, is associated with tumor metastasis and progression in mouse models of breast carcinoma^43^,^44^. Expression of Mena^INV^ in breast carcinoma cells increases invadopodium lifetime and degradative activity^45^.

Quantitative immunofluorescence assays of the expression of invasive Mena isoforms relative to total Mena expression (Mena^Calc^) have been developed to predict metastatic risk in patients with breast cancer^46^,^47^. Mena is expressed in tumor cells within sites of macrophage-contact-dependent tumor cell intravasation, termed Tumor Microenvironment of Metastasis (TMEM). The density of TMEM sites in patient tumor samples is an independent predictor of metastasis that positively correlates with Mena^INV^ expression^44^,^48^,^49^,^50^. Furthermore, increased Mena^INV^ protein expression, detected using a Mena^INV^-specific antibody is associated with increased recurrence and decreased survival in breast cancer patients^39^. In contrast, expression of Mena with the 11a exon is reduced in the invasive and disseminating subpopulation of breast carcinoma cells^40^. This is interesting because expression of Mena11a is associated with suppression of invasion and dissemination^48^,^51^.

Over-expression of Mena^INV^ produces tumor cells that are less cohesive and demonstrate increased invasion, intravasation and lung metastasis in mouse models of breast cancer^44^,^45^,^51^. Mena^INV^ also drives assembly of ECM networks with increased abundance of linear collagen fibrils radiating from the tumor periphery^39^. While over-expression of Mena lacking the INV exon also increases invasion and metastasis, the effects of Mena^INV^ expression are significantly more potent^45^. Despite the close relationship between Mena^INV^ expression and tumor invasiveness, the mechanism underlying Mena^INV^–dependent enhancement of invadopodium stability and function has not been explored.

Recent investigation into how Mena^INV^ contributes to tumor cell invasion and dissemination has focused on the ability of Mena^INV^ to sensitize invading breast cancer cells to chemotactic or haptotactic signals such as EGF or fibronectin present in the ECM^39^,^43^,^45^,^51^. Hughes *et al.*^43^ showed that Mena recruits the tyrosine phosphatase PTP1B to the EGF receptor (EGFR), but the Mena^INV^ isoform has the unique effect of inhibiting PTP1B recruitment to the EGFR^43^. Mena^INV^ also binds to α5 integrin more robustly than Mena^39^. These findings reveal that inclusion of the INV sequence influences interactions of Mena with specific effector molecules. The ability of Mena^INV^ to regulate PTP1B at the EGFR opens the possibility that Mena^INV^ may promote invasion through affecting PTP1B in a variety of other contexts in which the phosphatase acts as a tumor suppressor^52^. For example, PTP1B has been shown to bind and dephosphorylate cortactin at tyrosine 421 in human cells^53^,^54^, though the effect of PTP1B on invadopodium maturation has not been explored. Phosphorylation of cortactin at tyrosine 421 has been shown to occur at both invadopodia and the leading edge^55^, and is essential for both actin polymerization^17^ and ECM degradation at invadopodia^56^,^57^. While Mena^INV^ is known to increase EGF-elicited phosphorylation of cortactin at tyrosine 421^43^, whether this increase in cortactin tyrosine 421 phosphorylation occurs within specific subcellular compartments, and how this contributes to tumor cell phenotype, have not been determined.

In this study, we investigate the specific effects of the Mena^INV^ isoform on invadopodium biology, characterize the localization and arrival dynamics of Mena^INV^ at developing invadopodia, and investigate how Mena^INV^ may promote cell invasion through a direct effect on invadopodium maturation. Furthermore, we explore for the first time the role of PTP1B in regulating invadopodium maturation via its effect on cortactin phosphorylation at invadopodia, and address the possibility that Mena^INV^ interferes with this function of PTP1B at invadopodia.

## Results

### Mena isoforms localize to invadopodia in breast carcinoma cells

**Figure 1:**
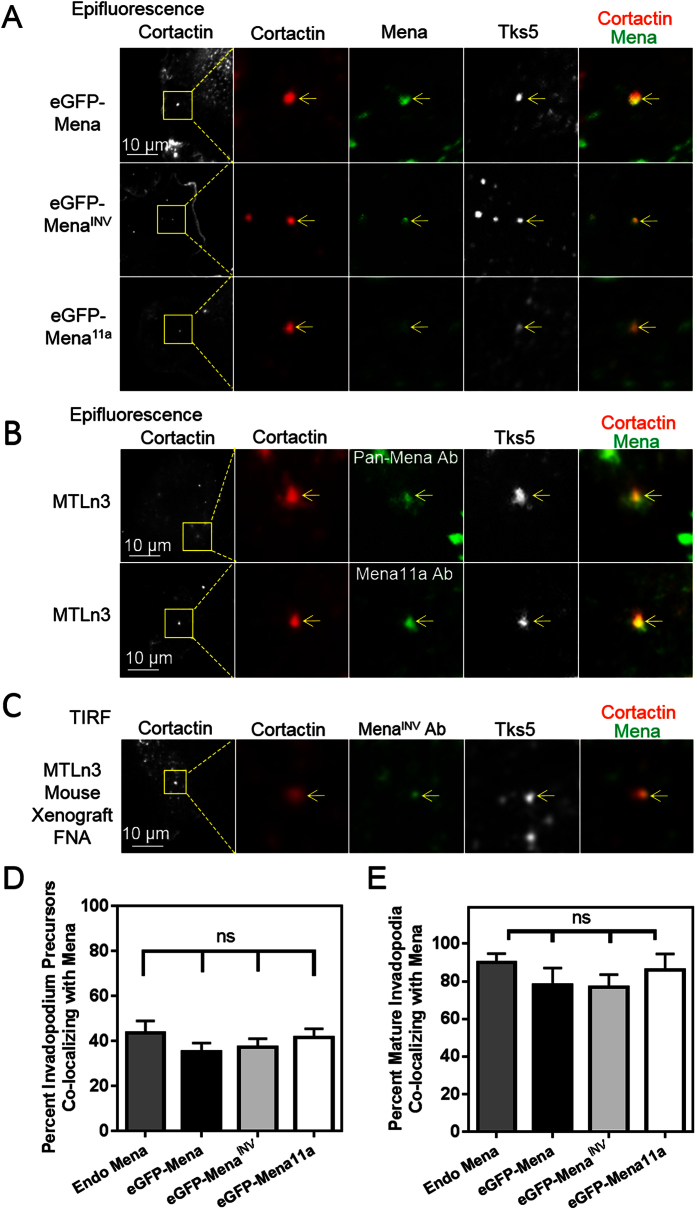
Mena^INV^ localizes to invadopodia in breast tumor cells. (**A)** Mena isoforms localize to invadopodium precursors. Representative images of MTLn3 cells expressing eGFP-Mena, eGFP-Mena^INV^ or eGFP-Mena11a showing co-localization of Mena isoforms with invadopodium markers. The cells were plated on fluorescently-labeled gelatin in complete media, fixed and immunostained for Tks5 and cortactin. Yellow arrows indicate regions of co-localization in all 3 channels depicted (n = three independent experiments). (**B)** Endogenous Mena localizes to the invadopodium. Representative images of MTLn3 cells showing co-localization of Mena, using a pan-Mena primary antibody that recognized all Mena isoforms, or Mena11a, using a Mena11a-specific antibody (Supplementary Fig. S2), with invadopodium markers. The cells were plated on unlabeled gelatin in complete media, fixed and stained for cortactin, Tks5, all Mena isoforms (Pan-Mena) and Mena11a (n = three independent experiments). (**C)** Mena^INV^ localizes at the invadopodium in tumor cells collected from mammary tumors by fine needle aspiration. Representative images of MTLn3 cells isolated from a mouse xenograft tumor three weeks after transplantation *in vivo* (described in the Methods section). The isolated cells were plated on unlabeled gelatin in complete media, fixed and immunostained for Mena^INV^, cortactin and Tks5 (n = three independent experiments). (**D)** The percentage of invadopodium precursors, defined as cortactin- and Tks5-rich puncta, co-localizing with eGFP-tagged Mena, Mena^INV^, Mena11a or endogenous Mena (Pan-Mena stain) in MTLn3 cells as depicted in (**A,B**). (**E)** The percentage of mature invadopodia co-localizing with eGFP-tagged Mena, Mena^INV^, Mena11a or endogenous Mena in MTLn3 cells as depicted in A-B. (n > 50 cells; three independent experiments). There were no statistical differences between group means as determined by one-way analysis of variance (ANOVA) and Tukey’s multiple comparisons test. ns = non-significant.

Previous work has shown that Mena localizes to invadopodia in mammary carcinoma cells^45^. However, the differences in localization of different Mena isoforms (Mena^INV^ and Mena11a) to invadopodia have not been assessed. Using both rat MTLn3 and human MDA-MB-231 breast carcinoma cell lines stably expressing eGFP-Mena, eGFP-Mena^INV^ and eGFP-Mena11a, we found that all of these Mena isoforms co-localize with cortactin and Tks5 at invadopodium precursors (Fig. 1A; Supplementary Fig. S1). These eGFP-tagged Mena, Mena^INV^ and Mena11a isoforms localize to approximately 40% of invadopodium precursors, with no significant difference in localization between the two isoforms (p = 0.8695, Fig. 1D).

We investigated whether endogenous Mena localization matched that seen in cells overexpressing eGFP-tagged Mena isoforms by staining parental MTLn3 cells with antibodies recognizing all Mena isoforms (Pan-Mena Ab) (Fig. 1B) and found that endogenous Mena localized to a similar proportion of invadopodium precursors (Fig. 1D). When we stained MTLn3 cells with a newly developed antibody specific to Mena11a (Supplementary Fig. S2E), we found that Mena11a localization to invadopodium precursors was similar to that of endogenous Mena (Fig. 1B). Since MTLn3 cells do not express detectible levels of Mena^INV^ protein in culture^44^,^58^ but do so when growing *in vivo*^40^, we collected and cultured fine needle aspiration (FNA) samples from mouse MTLn3 xenograft tumors which are known to spontaneously express Mena^INV^ in the tumor microenvironment^40^,^48^. These FNA-collected cells were stained with an antibody specific to Mena^INV^^59^. Mena^INV^ staining was detectible in a subset of the FNA cells (5–10%), where we found that Mena^INV^ localizes to invadopodia, as judged by overlap with cortactin and Tks5 immunofluorescence (Fig. 1C).

### Mena^INV^, but not Mena11a, promotes invadopodium maturation in breast carcinoma cells

**Figure 2:**
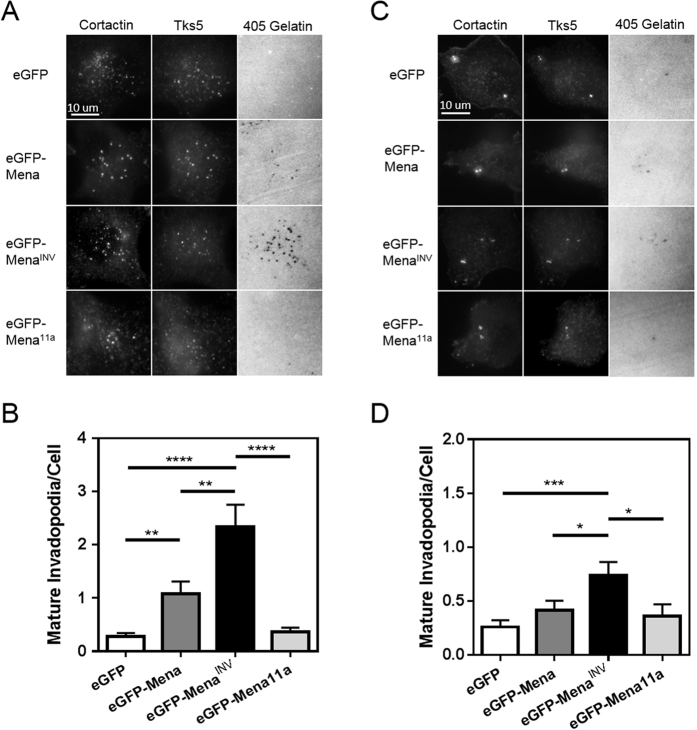
Mena isoforms expressed in invasive human and rat breast carcinoma cell lines have different effects on invadopodium maturation *in vitro.* (**A**) Mena^INV^ expression promotes invadopodium maturation in MDA-MB-231 human breast carcinoma cell lines. Representative images of MDA-MB-231 cells expressing eGFP, eGFP-Mena, eGFP-Mena^INV^, or eGFP-Mena11a plated on Alexa Fluor 405 labeled gelatin in complete media and immunostained for Tks5 and cortactin (n = three independent experiments). (**B**) Mena^INV^ expression promotes invadopodium maturation in MDA-MB-231 cells. Quantification of mature invadopodia in cells expressing the Mena isoforms listed in (**A**). Mature invadopodia were scored as the co-localization of Tks5- and cortactin-rich puncta with a degradation hole in the Alexa Fluor 405-labeled gelatin. (n > 90 cells/condition; n > 30 fields/condition; three independent experiments). (**C**) Mena^INV^ expression promotes invadopodium maturation in MTLn3 rat breast carcinoma cells. Representative images of MTLn3 cells expressing eGFP, eGFP-Mena, eGFP-Mena^INV^, and eGFP-Mena11a as in (**A**). (**D**) Mena^INV^ expression promotes invadopodium maturation in MTLn3 cells. Quantification of mature invadopodia as scored in B (n > 90 cells/condition; n > 30 fields/condition, three independent experiments). Error bars indicate ± SEM. Data was analyzed for statistical significance by unpaired t-test with Welch’s correction for unequal variance. *p < 0.05; **p < 0.01; ***p < 0.001; ****p < 0.0001.

It has previously been shown that Mena and Mena^INV^ expression increases extracellular matrix (ECM) degradation in rat breast carcinoma cells^45^. Since Mena^INV^ expression has been shown to drive tumor cell invasion, while Mena11a expression is associated with reduced invasion^44^,^45^,^51^, we assessed the effect of Mena, Mena^INV^ and Mena11a specifically on invadopodium maturation. We measured the number of mature invadopodia, as determined by the co-localization of invadopodia with holes in a fluorescently labeled gelatin matrix, in MDA-MB-231 and MTLn3 cells. We overexpressed eGFP-Mena, eGFP-Mena^INV^ and eGFP-Mena11a in MDA-MB-231^43^ and MTLn3^44^ cells at approximately 4-fold the level of endogenous Mena expression to simulate the level of spontaneous Mena^INV^ expression seen previously in the invasive subpopulations of breast carcinoma cells *in vivo* in two different mouse mammary tumors^40^. eGFP-Mena^INV^ expression increased the number of mature invadopodia per cell by approximately 8-fold in MDA-MB-231 cells and 3–4-fold in MTLn3 cells (Fig. 2). Expression of eGFP-Mena did not increase mature invadopodium number in MTLn3 cells (Fig. 2D), but eGFP-Mena did produce a significant increase in MDA-MB-231 cells (Fig. 2B). However the increase in mature invadopodium number produced by eGFP-Mena expression in MDA-MB-231 cells was significantly less than the increase produced by eGFP-Mena^INV^ (Fig. 2B). In contrast, Mena11a expression did not produce an increase in the number of mature invadopodia per cell in either cell line (Fig. 2).

**Figure 3:**
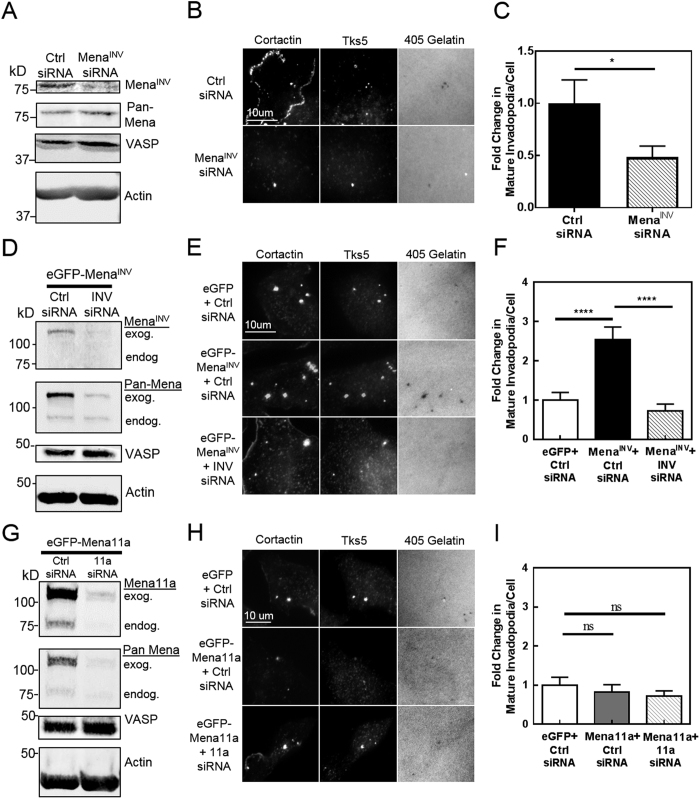
Reduction of Mena^INV^ expression limits invadopodium maturation. (**A**) Mena^INV^ knock-down (KD) in MDA-MB-231 cells. Western blot of lysates from MDA-MB-231 cells treated with siRNA targeting the INV exon of Mena or control siRNA for 72 hours, and stained with an antibody specific to Mena^INV^. VASP staining was used to control for off-target effects, and β-actin was used as a loading control. (**B**) Mena^INV^ KD reduces invadopodium maturation in MDA-MB-231 cells. Representative images of MDA-MB-231 cells from A) plated on Alexa Fluor 405 labeled gelatin in complete media, and immuno-stained for invadopodium markers. (**C**) Quantification of mature invadopodia in cells treated as in B). (n > 80 cells/condition; n > 30 fields/condition; three independent experiments). (**D**) Mena^INV^ KD in eGFP-Mena^INV^ expressing MTLn3 cells. Western blot of lysates from MTLn3 cells overexpressing eGFP-Mena^INV^, transfected with siRNA specifically targeting either the INV exon of Mena or control siRNA, and stained for all Mena isoforms (Pan-Mena) and Mena^INV^. VASP staining was used to control for off-target effects, and β-actin was used as a loading control. (**E)** Representative images of MLTn3 cells from (**D**) or expressing eGFP alone, plated on Alexa Fluor 405 labeled gelatin in complete media, and immuno-stained for invadopodium markers. (**F)** Quantification of mature invadopodia in cells treated as in E). (n > 90 cells/condition; n > 40 fields/condition; three independent experiments). (**G**) Mena11a KD in MTLn3 cells. Western blot of lysates from MTLn3 cells overexpressing eGFP-Mena11a, transfected with siRNA specifically targeting the 11a exon of Mena or control siRNA, and stained for all Mena isoforms (Pan-Mena) and Mena11a specifically. VASP staining was used to control for off-target effects and β-actin was used as a loading control. (**H**) Representative images of MTLn3 cells in (**G**) or expressing eGFP alone, plated on Alexa Fluor 405 labeled gelatin in complete media, immuno-stained for invadopodium markers as in (**B**). (**I**) Quantification of mature invadopodia per cell (Tks5- and cortactin-rich puncta co-localizing with degradation holes) of cells shown in H) (n > 90 cells/condition; n > 40 fields/condition; three independent experiments). Error bars indicate SEM. Data was analyzed for statistical significance by unpaired t-test with Welch’s correction for unequal variance. *p < 0.05; ****p < 0.0001; ns = non-significant.

Since MDA-MB-231 cells express low but detectible levels of endogenous Mena^INV^ *in vitro* (Fig. 3A)^60^, we used siRNA depletion of this endogenous Mena^INV^ to determine if it is necessary for normal invadopodium maturation in these cells. We used a siRNA specific to the INV exon of Mena to deplete this endogenous Mena^INV^ by more than 70% in MDA-MB-231 cells (Fig. 3A), and found that this decreased the level of mature invadopodia per cell by more than 50% (Fig. 3B,C). Since MTLn3 cells do not express endogenous Mena^INV^ (Fig. 3D)^44^, we transfected MTLn3 cells with eGFP-Mena^INV^ and found that the increase in mature invadopodia caused by Mena^INV^ overexpression was reversed when we depleted the level of exogenous Mena^INV^ expression by more than 80% using siRNA specific to the INV exon (Fig. 3D–F). In contrast, depletion of Mena11a expression by more than 80% using siRNA specifically targeting the 11a exon in MTLn3 cells overexpressing Mena11a did not have any effect on the number of mature invadopodia per cell (Fig. 3G–I). These results indicate that Mena^INV^ specifically promotes invadopodium maturation while Mena11a has no effect.

### Mena^INV^ localizes to invadopodium precursors prior to their stabilization and accumulates at mature invadopodia

Having shown that expression of Mena^INV^, but not Mena11a, increases invadopodium maturation, we next asked whether there were significant differences in the localization of these Mena isoforms to mature invadopodia. In MTLn3 cells stably expressing eGFP-tagged Mena, Mena^INV^ or Mena11a, each of these Mena isoforms localized to approximately 80% of mature invadopodia (Fig. 1E). There were no significant differences between eGFP-Mena, eGFP-Mena^INV^, eGFP-Mena11a or staining for endogenous Mena (Pan-Mena Ab) in percent localization to mature invadopodia (Fig. 1E). Endogenous Mena, eGFP-Mena, eGFP-Mena^INV^ and eGFP-Mena11a all localized to a significantly higher proportion of mature invadopodia than invadopodium precursors (Supplementary Fig. S2). Therefore, while each Mena isoform is able to localize to invadopodium precursors before ECM degradation begins, their localization is substantially enriched in mature invadopodia (Fig. 1D,E; Supplementary Fig. S2). Thus, although all Mena isoforms localize similarly to mature invadopodia, Mena^INV^ and Mena11a produce different effects on the maturation process.

**Figure 4:**
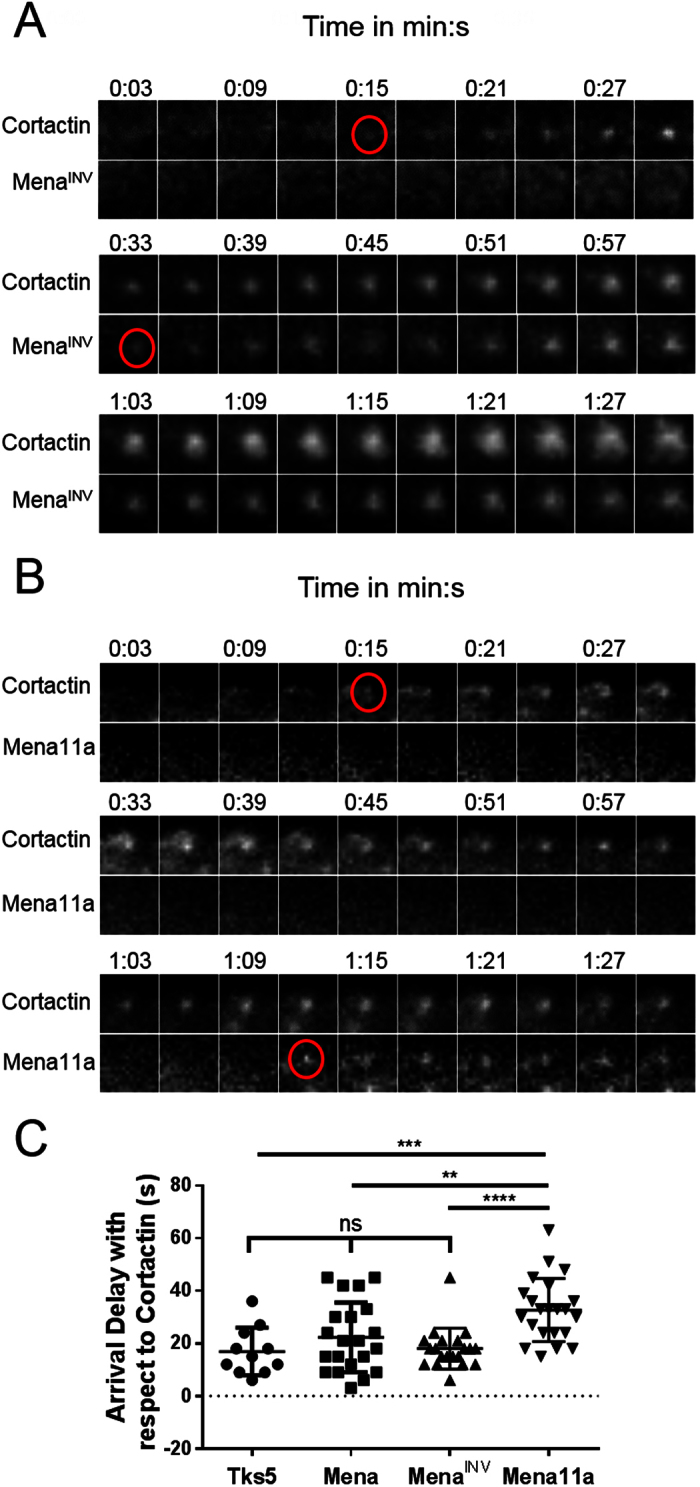
Mena^INV^ arrives at invadopodium precursors after core complex assembly, but before invadopodium stabilization at the plasma membrane. Time-lapse montage of TagRFP-Cortactin and (**A**) eGFP-Mena^INV^ or (**B**) eGFP-Mena11a in MTLn3 cells starved for 3 h and stimulated with 5 nM EGF during imaging. (**C**) Quantification of Mena, Mena^INV^ and Mena11a arrival delays with respect to cortactin. For comparison, the arrival delay of eGFP-Tks5 is also shown. Red lines indicate mean with 95% confidence interval. n = 21 invadopodia (Mena), 20 invadopodia (Mena^INV^), 22 invadopodia (Mena11a) and 11 invadopodia (Tks5). **p < 0.01; ***p < 0.001; ****p < 0.0001.

To examine the role of Mena^INV^ in the stages of invadopodium precursor assembly and invadopodium maturation in more detail, we next measured the arrival kinetics of Mena, Mena^INV^ and Mena11a relative to the arrival of established markers of the invadopodium at various stages of maturation^20^. Using time-lapse imaging of live MLTn3 cells expressing TagRFP-cortactin and eGFP-Mena, eGFP-Mena11a or eGFP-Mena^INV^, we found that Mena and Mena^INV^ co-localized with cortactin at newly-forming invadopodium precursors an average of 21 and 18 seconds after cortactin arrival, respectively, while Mena11a arrived slightly later, approximately 32 seconds after cortactin (Fig. 4C). This arrival delay relative to cortactin indicates that Mena^INV^ is recruited to the invadopodium precursor at the same time as Tks5 (Fig. 4C)^20^. Thus, Mena^INV^ arrives at the invadopodium precursor during a period prior to invadopodium maturation that is associated with Tks5-dependent stabilization of the precursor at the plasma membrane^20^. The arrival of Mena^INV^ during this period of invadopodium stabilization and actin polymerization^16^ supports its role in directly promoting invadopodium maturation.

### Mena^INV^ expression increases cortactin phosphorylation at invadopodia

Having established both the presence of Mena^INV^ at invadopodium precursors, and an increase in invadopodium maturation in cells expressing Mena^INV^, we began to investigate how Mena^INV^ specifically affects invadopodium maturation. The tyrosine phosphorylation of cortactin by Arg kinase in response to EGFR and β1-integrin activation have been shown to be key signaling steps in the transition of the invadopodium from the late precursor stage to a mature structure capable of degradation^17^,^21^,^22^,^61^. We therefore determined whether Mena^INV^ was influencing the cortactin phosphorylation state within invadopodia differently from that occurring with Mena11a, thereby promoting invadopodium stability and maturation.

**Figure 5:**
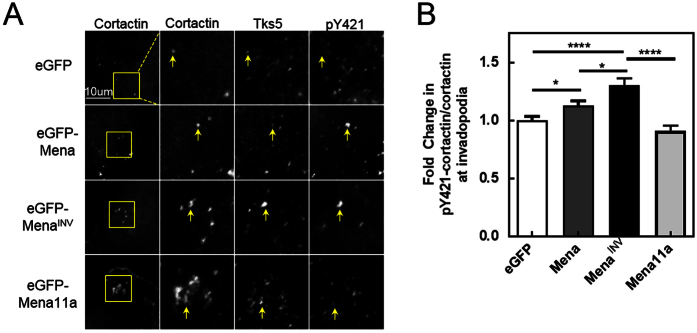
Mena^INV^ expression promotes cortactin phosphorylation and invadopodium maturation. (**A**) Representative images of MDA-MB-231 cells transduced with eGFP, eGFP-Mena, eGFP-Mena^INV^ or eGFP-Mena11a using a retrovirus and plated on thin gelatin matrix were starved overnight. Cells were fixed and stained for cortactin, phospho-Y421 cortactin, and Tks5. (**B**) Quantification of cortactin phosphorylation expressed as the fold change in pY421-cortactin/cortactin ratio localized to punctate invadopodium precursors, defined as punctate co-localization of cortactin and Tks5 from (**A**) (n > 200 invadopodia and 50 cells for each condition; three independent experiments). Error bars indicate ± SEM. Data was analyzed for statistical significance by unpaired t-test with Welch’s correction for unequal variance. *p < 0.05; ****p < 0.0001.

To determine whether Mena^INV^ influenced cortactin phosphorylation at invadopodia we assessed the level of tyrosine 421 cortactin phosphorylation in both human and rat breast carcinoma cells that were serum starved to reduce background levels of cortactin phosphorylation. Interestingly, we found that in MDA-MB-231 cells that were serum-starved Mena^INV^ expression produced a significant increase in cortactin phosphorylation at tyrosine 421 within invadopodia relative to the eGFP control. Mena11a expression, which did not promote invadopodium maturation, produced no significant effect on cortactin phosphorylation, as expected (Fig. 5). Expression of Mena without either the INV or 11a exons also produced an increase in cortactin phosphorylation at tyrosine 421 within invadopodia, but this increase was significantly less than that produced by Mena^INV^ expression (Fig. 5). Thus expression of Mena^INV^ and, to a lesser extent, Mena, but not Mena11a increases cortactin phosphorylation at tyrosine 421 independently of exogenous soluble growth factors, such as EGF or HGF. Given the known regulation of invadopodium maturation by cortactin phosphorylation, the ability of Mena^INV^ to increase cortactin phosphorylation at invadopodia may, at least in part, underlie Mena^INV^-driven increases in invadopodium maturation and matrix degradation^16^,^23^,^24^,^62^.

**Figure 6:**
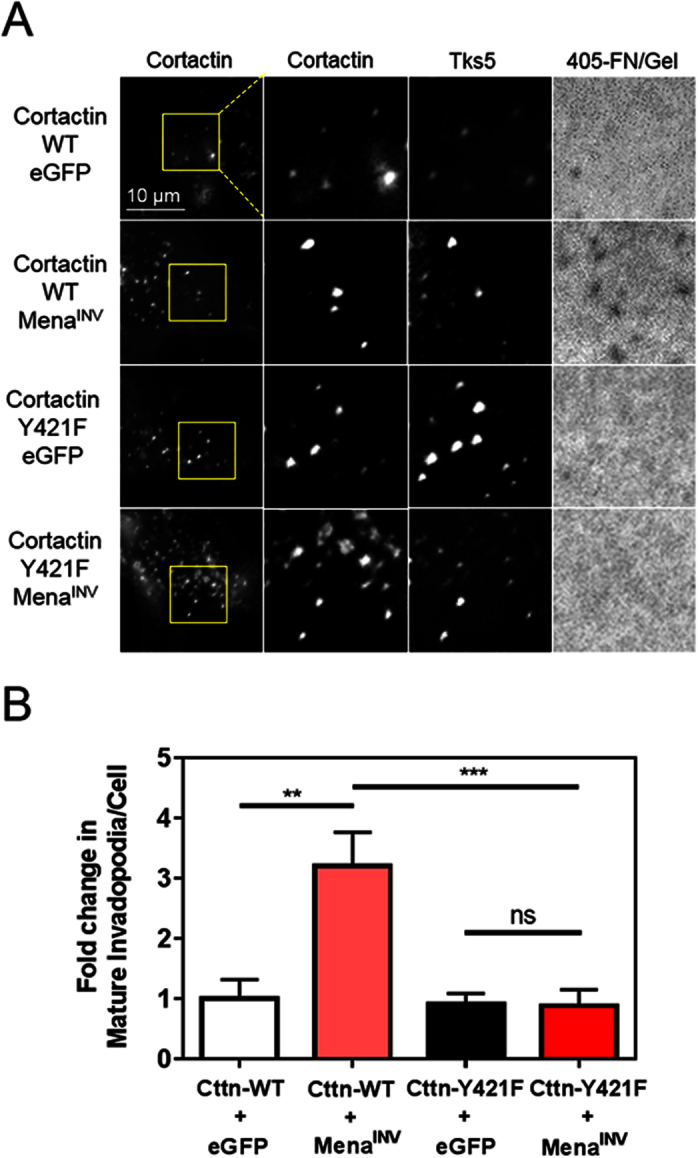
The increase in invadopodium maturation produced by Mena^INV^ is dependent on cortactin phosphorylation. (**A**) Representative images of MDA-MB-231 cells stably expressing wild-type cortactin or cortactin in which tyrosine 421 was mutated to phenylalanine (Y421F), with transient knock down of endogenous cortactin using siRNA and transient forced expression of eGFP-Mena^INV^ or an eGFP control vector. Cells were plated on Alexa Fluor 405 labeled FN/gelatin and stained for cortactin and Tks5. (**B**) Quantification of mature invadopodia, identified as cortactin- and Tks5-rich puncta co-localizing with a degradation hole, for the images shown in (**A**) (n < 50 cells; three independent experiments). Data was analyzed for statistical significance by unpaired t-test with Welch’s correction for unequal variance. **p < 0.01; ***p < 0.001; ns = not significant.

To determine if the increase in mature invadopodia produced by Mena^INV^ expression is dependent on the increase in cortactin tyrosine 421 phosphorylation, we used a non-phosphorylatable Y > F mutant of cortactin. If the increase in invadopodium maturation caused by Mena^INV^ expression is dependent on cortactin phosphorylation, then cells expressing the phosphorylation-deficient mutant of cortactin should not produce additional mature invadopodia when Mena^INV^ is overexpressed. MDA-MB-231 cells stably expressing wild-type murine cortactin (cortactin-WT) or a cortactin mutant incapable of being phosphorylated at tyrosine 421 (cortactin-Y421F), were each depleted of endogenous human cortactin (Supplementary Fig. S3) and transfected with eGFP or eGFP-Mena^INV^. Quantification of the number of mature invadopodia that formed after plating the cells on a thin fluorescent ECM indicated that Mena^INV^ expression increased the number of mature invadopodia in cells expressing cortactin-WT by 3–4-fold, but produced no change in the number of mature invadopodia in cells expressing cortactin-Y421F (Fig. 6). Therefore the increase in invadopodium maturation due to Mena^INV^ expression is dependent upon cortactin phosphorylation at tyrosine 421.

### PTP1B regulates cortactin phosphorylation at invadopodia and opposes the effect of Mena^INV^

Cortactin is phosphorylated at tyrosine 421 in invadopodia by Arg, a tyrosine kinase that is activated by a variety of upstream signals, including EGF stimulation and β1-integrin activation^21^,^22^,^62^,^63^. However the process by which cortactin tyrosine 421 is dephosphorylated at invadopodia, and invadopodium maturation is thereby regulated, has not been studied. During hyperosmolarity-induced stress, cortactin phosphorylation at tyrosine 421 and 446 has been shown to be regulated by the non-receptor protein tyrosine phosphatase PTP1B^53^. Furthermore, cortactin phosphorylation at tyrosine 421 was upregulated nearly 5-fold in a quantitative mass-spectroscopy analysis of phospho-tyrosines in PTP1B deficient mice^54^. An analysis of a large cohort of histological samples from breast cancer patients showed that PTP1B expression is associated with less aggressive, estrogen-receptor-positive disease, and overall greater survival^64^. Interestingly, pharmacological inhibition of PTP1B activity has been shown to produce higher rates of breast carcinoma cell invasion *in vitro* as well as *in vivo*^43^. We therefore investigated whether PTP1B expression has an additional role in suppressing tumor invasion by regulating the phosphorylation of cortactin Y421 at invadopodia.

**Figure 7:**
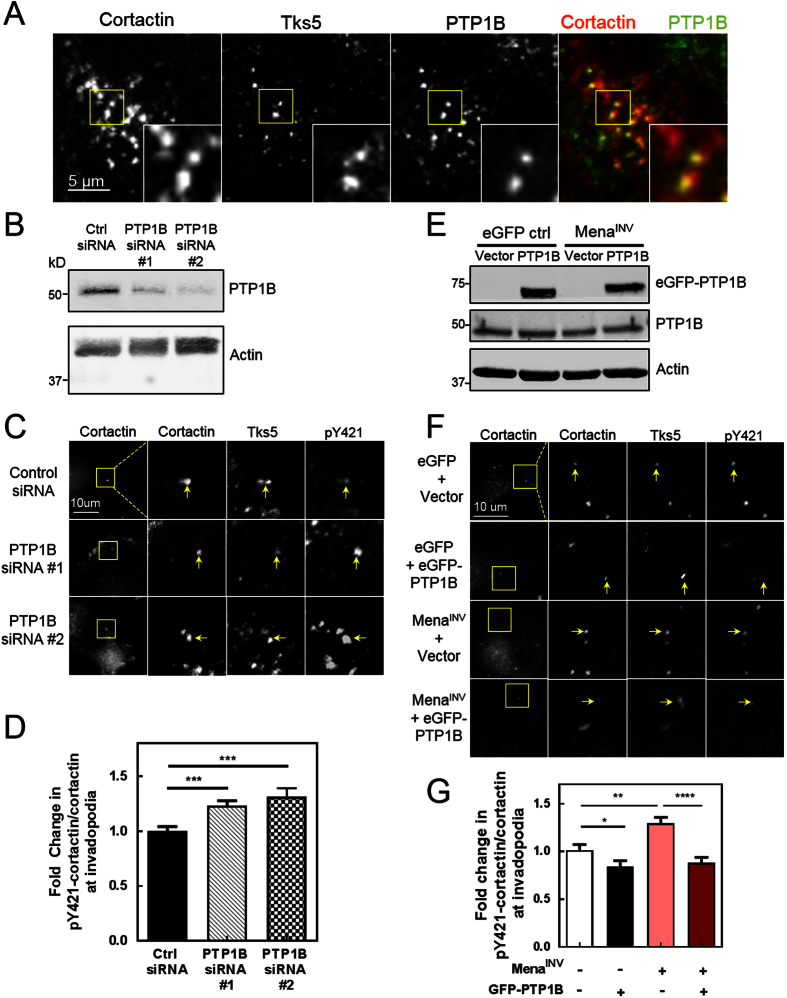
PTP1B localizes to invadopodium precursors, where it suppresses pY421-cortactin phosphorylation and reverses Mena^INV^-induced cortactin phosphorylation. (**A**) Representative images of MDA-MB-231 cells immuno-stained for cortactin, Tks5 and PTP1B, imaged on a TIRF microscope showing PTP1B localizing to invadopodium precursors. (**B**) Western blot showing MDA-MB-231 lysates treated with control or one of two PTP1B-targeting single siRNAs for 48 h and stained for PTP1B using a monoclonal antibody (n = three independent experiments). (**C**) PTP1B KD increases Y421-cortactin phosphorylation. Representative images of MDA-MB-231 cells transfected with control or one of two PTP1B siRNAs for 48 h, plated on 0.2% gelatin matrix and starved overnight. Cells were then fixed and stained for cortactin, phospho-Y421 cortactin, and Tks5. (**D**) Cortactin phosphorylation expressed as the fold change in pY421-cortactin/cortactin ratio localized to invadopodium precursors, defined as punctate co-localization of cortactin and Tks5 (n < 100 invadopodia; three independent experiments). (**E**) eGFP-PTP1B overexpression in MDA-MB-231 cells. Western blot showing lysates from MDA-MB-231 cells stably expressing eGFP or eGFP-Mena^INV^ were transfected with 0.5 ug of an empty-vector control or pEGFP-PTP1B-HA C3 DNA and stained for PTP1B. (n = three independent experiments) (**F**) eGFP-PTP1B overexpression suppresses basal and Mena^INV^-increased Y421-cortactin phosphorylation. Representative images from cells in (**E**), after fixation and staining for pY421-cortactin, cortactin and Tks5. (**G**) eGFP-PTP1B overexpression suppresses basal and Mena^INV^-increased Y421-cortactin phosphorylation. Quantification of the fold change in pY421-cortactin/cortactin ratio localized to invadopodium precursors, defined as punctate co-localization of cortactin and Tks5 (n > 100 invadopodia; three independent experiments). Error bars indicate ± SEM. Data was analyzed for statistical significance by unpaired t-test with Welch’s correction for unequal variance. *p < 0.05, **p < 0.01; ***p < 0.001; ****p < 0.0001.

**Figure 8:**
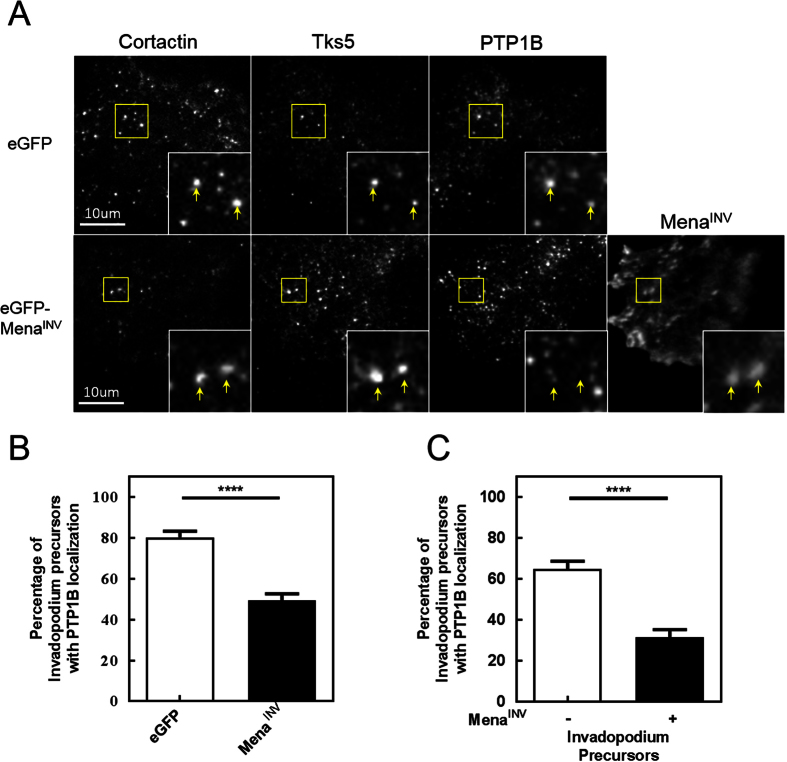
Mena^INV^ reduces PTP1B localization to invadopodium precursors. (**A**) Representative images of MDA-MB-231 cells expressing eGFP or eGFP-Mena^INV^ and immuno-stained for cortactin, Tks5 and PTP1B, imaged on a TIRF microscope. (**B**) The percentage of invadopodium precursors co-localizing with PTP1B staining was quantified from the images shown in (**A**) (n > 50 cells). (**C**) Invadopodium precursors from MDA-MB-231 cells expressing eGFP-Mena^INV^ in the images shown in (**A**) were divided into those with (+) or without (−) Mena^INV^ localization, and the proportion of each population with PTP1B localization was quantified (n > 50 cells). Error bars indicate ± SEM. Data was analyzed for statistical significance by unpaired t-test with Welch’s correction for unequal variance. ****p < 0.0001.

We found that PTP1B localizes to more than 50% of invadopodium precursors in both MDA-MB-231 and MTLn3 cells (Fig. 7A; Supplementary Fig. S4). PTP1B localized to approximately 80% of invadopodium precursors in MDA-MB-231 cells expressing eGFP alone, but when eGFP-Mena^INV^ was overexpressed PTP1B localization to invadopodium precursors dropped from 80% to less than 50% (Fig. 8A,B). When we compared invadopodium precursors that contained eGFP-Mena^INV^ with those that did not, we found that significantly fewer invadopodia containing Mena^INV^ also contained PTP1B (Fig. 8C), indicating that Mena^INV^ and PTP1B localization to invadopodia are inversely correlated. Reduction of PTP1B expression with siRNA, to less than 20% of control levels (Fig. 7B; Supplementary Fig. S4), increased the level of cortactin phosphorylation at tyrosine 421 within invadopodia of both MDA-MB-231 (Fig. 7C,D) and MTLn3 (Supplementary Fig. S4) cells to a similar extent as Mena^INV^ overexpression. Knock down of PTP1B expression by siRNA also significantly increased the number of mature invadopodia per cell and the level of matrix degradation per invadopodium (Supplementary Fig. S4).

We further interrogated the role of PTP1B in regulation of cortactin phosphorylation in invadopodia using forced-expression of exogenous PTP1B (eGFP-PTP1B-HA). We found that eGFP-PTP1B-HA expression in both MDA-MB-231 and MTLn3 cells decreased cortactin tyrosine 421 phosphorylation at invadopodia relative to cells expressing eGFP alone (Fig. 7E–G; Supplementary Fig. S4), and eGFP-PTP1B-HA expression in MTLn3 cells significantly reduced the number of mature invadopodia per cell relative to eGFP alone (Supplementary Fig. S4). Furthermore, PTP1B forced-expression in cells stably expressing eGFP-Mena^INV^ reversed the increase in tyrosine 421 cortactin phosphorylation at invadopodia caused by eGFP-Mena^INV^ relative to cells expressing eGFP alone (Fig. 7G). Thus, PTP1B regulates cortactin phosphorylation at tyrosine 421 in invadopodium precursors of both rat and human breast carcinoma, limiting invadopodium maturation. While Mena^INV^ reduces PTP1B localization to invadopodium precursors, decreases cortactin phosphorylation at tyrosine 421, and promotes invadopodium maturation.

## Discussion

**Figure 9:**
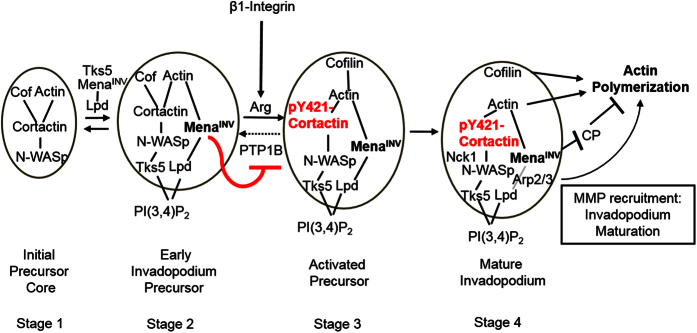
Model of Mena^INV^ recruitment to and signaling at invadopodia. Mena^INV^ arrives early during invadopodium precursor assembly at approximately the same time as Tks5 arrival. Tks5 begins stabilization of the precursor by binding it to sites of PI(3,4)P2 at the plasma membrane^20^. This is consistent with the recruitment of Mena and Tks5 to the plasma membrane at PI(3,4)P2-rich sites by Lamellipodin (Lpd)^20^,^35^. Mena^INV^ then displaces PTP1B from the invadopodium precursor (red line), preventing PTP1B from dephosphorylating cortactin at tyrosine 421 (dotted arrow). Y421-cortactin (red) phosphorylation leads to cofilin release and actin severing to produce a greater concentration of barbed ends. Cortactin phosphorylation also recruits Nck1 to the invadopodium, which acts through N-WASP to produce greater levels of Arp2/3-mediated actin branching from newly elongating barbed ends produced by cofilin severing^25^. Mena promotes actin polymerization by displacing Capping Protein (CP) from the barbed end, and Mena^INV^ promotes additional actin polymerization at invadopodia through enhanced cortactin phosphorylation at tyrosine 421, as described above, which leads to invadopodium elongation and maturation.

Our previous work has demonstrated the role of Mena^INV^ as a central regulator of breast carcinoma cell invasion, ECM remodeling, intravasation, and metastasis^39^,^40^,^44^,^45^,^48^,^51^. We have also established that Mena^INV^ expression is significantly higher in metastatic primary breast tumors^58^, correlates with disease recurrence and outcome in breast cancer patients^39^ and correlates with TMEM, a prognostic marker for metastasis in ER + /HER2- breast cancer patients^44^,^48^,^49^,^50^. In this study we further elucidate the relationship between Mena^INV^ and breast carcinoma cell invasion and dissemination by focusing on the effect of Mena isoform expression on invadopodium maturation, important because the invadopodium is a protrusive structure essential for intravasation^9^,^15^,^48^. Based on the findings presented here, we propose a model in which Mena^INV^ localizes to the invadopodium precursor and increases cortactin phosphorylation at tyrosine 421 to promote invadopodium maturation by interfering with PTP1B (Fig. 9).

In particular, we found that the effect of Mena^INV^ on increasing invadopodium maturation is specific to the INV isoform and that Mena and Mena11a did not show similar effects. It should be noted that previously it was reported that exogenous Mena expression increases the level of ECM degradation in MTLn3 cells^45^, while here we have found that exogenous Mena alone does not produce a significant increase in the number of mature invadopodia per cell. Since degradation area is not directly related to the number of mature invadopodia in a cell but rather is a measurement also related to the extent of cell motility over the ECM degradation target, we used in our study direct measures of mature invadopodium number to avoid misinterpretation of the effects of Mena isoform expression on invadopodium maturation. We found that Mena^INV^ localizes to invadopodium precursors during the period in which they are stabilized at the plasma membrane^20^, before peak cortactin phosphorylation occurs^17^. We provide evidence that PTP1B regulates cortactin phosphorylation at tyrosine 421 in invadopodia. Our current results showing that Mena^INV^ suppresses the activity of PTP1B toward cortactin within invadopodia are consistent with our previous work showing that, in EGF stimulated cells, Mena^INV^ suppresses the activity of PTP1B towards cortactin and other substrates^43^.

The phosphorylation of cortactin tyrosine 421 at invadopodia is necessary for the release of cofilin from cortactin, and recruitment of an Nck1/N-WASP/Arp2/3 complex, both of which are essential for actin-polymerization at the invadopodium precursor, and necessary to support cofilin-dependent actin polymerization that is required for invadopodium maturation into a matrix-degrading structure^17^,^22^,^23^,^24^,^25^. Our finding that Mena^INV^ is able to increase tyrosine 421 cortactin phosphorylation at invadopodia in the absence of exogenously-added soluble growth factors, such as EGF, raises the possibility that other cues, such as contact with the ECM can trigger this effect in invadopodia. β1 integrin binds to and activates Arg at invadopodia, leading to enhanced cortactin phosphorylation at tyrosine 421, and β1 integrin activity is necessary for invadopodium maturation^61^,^62^,^63^. Mena forms an adhesion-regulated complex with α5β1 integrin, a fibronectin receptor^38^, and Mena^INV^ enhances the ability of cells to migrate up fibronectin gradients present near the vasculature^39^. In limiting PTP1B’s regulation of cortactin phosphorylation at invadopodia, Mena^INV^ may sensitize carcinoma cells to produce mature invadopodia in response to a variety of β1-integrin ligands present in the tumor microenvironment, such as fibronectin and type-1 collagen^65^,^66^,^67^.

By upregulating Mena^INV^ expression, tumor cells may become more versatile in their ability to produce stable, mature invadopodia in a variety of microenvironments, including those where insoluble ligands are predominantly present to stimulate invadopodium maturation such as at the basement membranes of blood vessels. Fibronectin in particular has been shown to be abundant in the tumor stroma and fibronectin expression has been shown to correlate positively with tumor aggressiveness and decreased survival^68^,^69^,^70^,^71^. Even before tumor cells are exposed to soluble chemoattractants released from the vasculature, Mena^INV^ expression would allow the cells to invade through the fibronectin-rich basement membrane and surrounding stroma by producing more stable invadopodium protrusions. Mena^INV^-induced increases in haptotaxis and matrix degradation in response to insoluble ligands provide additional mechanisms by which metastatic cancer cells may resist EGFR inhibitor treatment^72^.

### Mena isoforms show similar localization to invadopodia, but have different effects on invadopodium signaling

The INV exon is located just downstream of Mena’s EVH1 domain. The EVH1 domain is responsible for Mena localization to the leading edge, focal adhesions and filopodia via binding to proteins containing a consensus sequence (F/WPXøP, X = any residue, ø = hydrophobic residue)^30^,^33^,^37^. Here we have found that eGFP-tagged Mena, Mena^INV^, Mena11a, and endogenous Mena isoforms, localize to a similar proportion of invadopodium precursors. Exogenous Mena, Mena^INV^ and Mena11a arrive at invadopodium precursors within seconds of each other, but Mena^INV^ was able to increase invadopodium maturation to a much greater extent than either of the other isoforms. Though Mena11a arrives at invadopodium precursors an average of ~14s later than Mena^INV^, all three isoforms arrived well before the minimum two-minute lag-time between precursor formation and matrix degradation. Furthermore, the slightly later arrival of Mena11a at invadopodium precursors does not explain its inability to promote cortactin phosphorylation at invadopodia in steady-state conditions, unlike Mena^INV^ expression (Fig. 5; Supplementary Fig. S3). These results suggest that the INV exon does not significantly alter Mena localization to invadopodia, but rather affects Mena function at invadopodia. PTP1B also contains a domain (LEPPPEHIPPPP) resembling the consensus EVH1 binding motif, and has been shown to bind to Mena containing an intact EVH1 domain^43^. Mena^INV^ expression has been shown to prevent PTP1B association with the EGFR^43^, and here we show that Mena^INV^ decreases association of PTP1B with invadopodia (Fig. 8). Hence, our data are consistent with the speculation that the INV exon enables Mena to disrupt the association of PTP1B with its substrates and this has the consequence of increasing invadopodium maturation by interfering with the proximity of PTP1B to cortactin at the invadopodium, thereby suppressing cortactin tyrosine dephosphorylation at that site.

Interestingly, we found that eGFP-Mena expression also increases invadopodium maturation in MDA-MB-231 cells (Fig. 2A,B), which endogenously express detectible Mena^INV^ *in vitro*^60^. However, eGFP-Mena expression did not produce a significant increase in invadopodium maturation in MTLn3 cells (Fig. 2C,D), which do not express detectible Mena^INV^ 44. Ena/VASP proteins, including Mena, have been shown to form hetero- and homotetramers via their EVH2 domains^42^,^73^,^74^. Since co-expressed Mena isoforms can associate in heterotetramers a given Mena isoform may thereby influence the localization and stability of other Mena isoforms that are co-expressed. Such heterotetramers may therefore allow exogenous Mena to recruit and/or stabilize endogenous Mena^INV^ at invadopodia in MDA-MB-231 cells. Thus, Mena expression may promote invadopodium maturation through its association with Mena^INV^ in tetramers, yet this would not be expected to occur in cell lines that do not express Mena^INV^ endogenously.

Another possible reason why Mena overexpression can promote invadopodium maturation is through its effects on actin polymerization^45^. It seems plausible that Mena^INV^ has a specific role in displacing PTP1B while increased levels of Mena (and/or Mena^INV^) would enhance actin polymerization. However, this seems less likely since the switch to invadopodium maturation precedes cofilin-dependent actin polymerization (Fig. 9) suggesting that the effect provided by endogenous Mena^INV^ on cortactin phosphorylation is the dominant factor in invadopodium maturation. Additional studies into the effects of co-expression and interaction of these Mena isoforms may yield valuable insights into their respective functions in tumor cells.

Though the Mena isoforms assessed in this study co-localized with the invadopodium markers Tks5 and cortactin, we found that the Mena isoform distributions at invadopodia were more diffuse than either of the two core markers Tks5 and cortactin. Mena isoforms have been shown to bind and be recruited by PH domain-containing proteins such as Lamellipodin^33^,^35^, and by SHIP2^75^ which in turn bind to phosphoinositides at the plasma membrane. The localization of the phosphoinositide PI(3,4)P2 to invadopodia has recently been characterized^20^, and localizes to invadopodia with the similarly diffuse distribution that we observe here for Mena isoforms (Fig. 1A–C; Supplementary Fig. S1). Mena is also known to bind the barbed end of actin filaments via its EVH2 domain^73^, and the localization of Mena to specific subcompartments of invadopodia defined by actin, phosphoinositides and adhesion proteins remains unclear. Answering this question will require higher-resolution imaging of the association of Mena isoforms with these invadopodum subcompartments, and may shed key insights into how Mena isoforms are recruited to and regulate invadopodia.

### PTP1B as a regulator of cortactin phosphorylation and invadopodium maturation

One of the key findings of our study is the identification of the phosphatase PTP1B as a regulator of cortactin phosphorylation at invadopodia in both rat and human breast carcinoma cells. This finding has implications concerning the consideration of PTP1B as a therapeutic drug target, and supports evidence that PTP1B expression is a potential prognostic marker in breast carcinoma patients^64^. Interest in the role of PTP1B in breast cancer progression began with the finding that PTP1B is overexpressed in human breast carcinoma cells transformed with the neu oncogene^76^ and overexpressed in the majority of breast tumors in patients relative to normal breast tissue^77^. However more recent analysis of tumors from larger cohorts of breast cancer patients indicates that PTP1B expression is associated with less aggressive, estrogen-receptor-positive disease, and overall greater survival^64^. In addition, a variety of substrates have been found for PTP1B that support its role as a tumor suppressor^52^.

Much of the evidence for PTP1B as a tumor promotor has come from its association with ErbB2-positive tumorigenesis^78^, though PTP1B’s role in later stages of tumor progression has received relatively little attention. Evidence for a pro-metastatic role for PTP1B has centered on PTP1B’s ability to promote Src kinase activity^79^,^80^, and PTP1B has previously been reported to promote, rather than suppress, invadopodium formation in breast cancer cells after being released from the ER membrane by calpain cleavage^81^,^82^. Cortesio *et al.*^81^ found that PTP1B dephosphorylation of Src kinase at tyrosine 529 increased Src activity, thereby promoting invadopodium formation. However, the increase in Src phosphorylation measured was at the whole cell level rather than specifically at invadopodia. Furthermore Cortesio *et al.*^81^, observed an increase in invadopodia (identified by cortactin and actin co-localization) but did not address the possibility that PTP1B may have distinct effects on invadopodium precursor initiation and the maturation of precursors to matrix-degrading structures as indicated in Fig. 9. Since cortactin has been found to co-localize with actin in endosomes, which appear as cortactin- and actin-rich cytoplasmic puncta^83^, actin-cortactin co-localization is not specific to the invadopodium core and not definitive to identify invadopodia. The cortactin and Tks5 markers used to identify the invadopodium core in our study co-localize only at invadopodia, and have highly divergent localization patterns outside of invadopodia. When we used cortactin and Tks5 as invadopodium markers in breast tumor cells, we found that knocking down PTP1B with siRNA increased invadopodium-associated ECM degradation and the number of mature invadopodia per cell (Supplementary Fig. S4). Furthermore, expression of exogenous PTP1B decreased the number of mature invadopodia per cell (Supplementary Fig. S4), indicating that PTP1B suppresses invadopodium maturation.

The discovery of Mena^INV^-dependent invadopodium maturation provides important new insight into the pro-metastatic effects of this isoform. Our results indicate that the effects of Mena^INV^ on tumor cell phenotype, including the sensitization of receptor tyrosine kinases (RTKs)^43^, invadopodium maturation, migration^51^ and dissemination^48^, result in part from the ability of Mena^INV^ to limit PTP1B-dependent dephosphorylation of RTKs and cortactin in tumor cells resulting in Mena^INV^-dependent invadopodium maturation which is required for tumor cell intravasation^9^,^10^,^15^. Furthermore, the discovery of Mena^INV^-dependent invadopodium maturation is consistent with the functional relationship between Mena isoform expression patterns and TMEM intravasation sites found in breast tumors^29^,^48^,^84^, two prognostics which are independently predictive of metastatic recurrence and mortality in breast cancer patients^29^,^46^,^47^,^49^,^50^.

## Methods

### Cell Lines and Cell Culture

MDA-MB-231 cells were cultured in DMEM (Life Technologies), supplemented with 10% FBS (Atlanta Biologicals, Flowery Branch, GA) and 0.5% penicillin/streptomycin, and MTLn3 cells were cultured in α-MEM supplemented with 5% FBS (Gemini Bio-Products, West Sacramento, CA) and 0.5% penicillin/streptomycin. In experiments involving serum starvation, MDA-MB-231 were starved overnight in DMEM containing 0.5% FBS and 0.8% BSA before adding L15 media supplemented with 0.345% BSA for an additional 3 h prior to fixation. MTLn3 cells were starved for 3–4 h in L15 media supplemented with 0.345% BSA prior to fixation, or stimulation with 5 nM mouse EGF (Life Technologies) during time-lapse imaging, as described previously^20^. EGF stimulation used during time-lapse imaging was performed in a homeostasis chamber maintained at 37 °C. MTLn3 and MDA-MB-231 cells stably expressing eGFP, eGFP-Mena, eGFP-Mena^INV^ or eGFP-Mena11a were created using retroviral vectors, with retroviral packaging, infection, and fluorescence activated cell sorting performed as described elsewhere^85^. MDA-MB-231 cells expressing murine cortactin and mutant cortactin-Y421F were generated using single-site directed mutagenesis of cortactin by PCR, cloning the mutated or wild-type construct into N1-TagRFP (Clontech Laboratories), and sub-cloning this into the retroviral expression vector pLSXN as described previously^22^.

### Fine Needle Aspiration of MTLn3 Xenografts

FNA was performed as described previously^44^ from SCID mice 3 weeks after injection of MTLn3 cells into the mammary gland. In brief, carcinoma cells were collected with a 25-gauge needle, centrifuged at low speed, resuspended in α-MEM containing 15% FBS, plated on 0.2% unlabeled gelatin for 3–4 h, washed 3 times in warm PBS to remove extracellular debris, and fixed in 3.7% paraformaldehyde. All procedures were conducted in accordance with the NIH regulations and approved by the Albert Einstein College of Medicine animal use committee.

### Antibodies, Drugs and DNA Constructs

The cortactin (ab33333), PTP1B EP1837Y (ab52650) and PTP1B EP1841Y (ab75856) antibodies were obtained from Abcam (Cambridge, MA). The Tks5 (sc30122) antibody was obtained from Santa Cruz Biotechnology (Santa Cruz, CA) and labeled with Alexa Fluor 568 dye (Molecular Probes, Eugene, OR) for pY421-cortactin staining experiments as per the manufacturer’s instructions. The phospho-Y421 cortactin (C0739) antibody was from Sigma-Aldrich (St. Louis, MO). The PTP1B inhibitor (539741) was from Calbiochem (San Diego, CA). All secondary antibodies were Alexa Fluor conjugated and obtained from Molecular Probes (Life Technologies, Carlsbad, CA). The Pan-Mena antibody was purchased from Novus Biologicals (Littleton, CO; Cat# NBP1–87914). The antibody specific for Mena^INV^ used in this study has been described elsewhere^59^. pEGFP-PTP1B C3 was a gift from Anna Huttenlocher (University of Wisconsin, Madison, WI). pCAX-eGFP and pCAX-eGFP-Mena^INV^ vectors were produced by the Gertler lab (MIT, Cambridge, MA)^45^.

### Production of Mena11a antibody

Rabbit polyclonal antibodies were generated by Covance. Animals were immunized with a peptide containing the sequence of the 11a exon of Mena11a.

Consistent with the specificity of this antibody, a western blot of MTLn3 cells containing eGFP-Mena11a contain a single band for endogenous Mena11a when stained with the anti-Mena11a IgG, corresponding in size with the band seen when the same blot was stained for all Mena isoforms (Pan-Mena), and a more intensely-staining band corresponding to eGFP-Mena11a (Fig. 3G) as has been described previously^44^. Western blots of lysates from cells previously characterized as containing Mena11a (MCF7)^86^ and parental MTLn3 cells also contained a band corresponding in size with endogenous Mena11a (Supplementary Fig. S2E). Additionally, western blots of cells obtained from mammary tumors in wild-type or Mena-null (Mena^−/−^) PyMT mice demonstrate the specificity of this antibody. Lysates obtained from WT PyMT tumor cells contain a band corresponding to endogenous Mena11a when stained with the anti-Mena11a IgG while lysates from Mena-null PyMT tumor cells do not contain a corresponding band (Supplementary Fig. S2E). All procedures were conducted in accordance with the NIH regulations and approved by the Albert Einstein College of Medicine animal use committee.

### RNA interference and transfection

Non-targeting siRNA (D-001210-01), siRNAs targeting human PTP1B (J-003529-15, 5′-CUACCUGGCUGUGAUCGAA-3′; J-003529-14, 5′-GGAGAAAGGUUCGUUAAAA-3′), siRNA targeting rat PTP1B (J-080071-10, 5′-GAUCGAGGGUGCAAAGUUC-3′) and siGENOME siRNA Smart Pool targeting human cortactin (M010508-00-0005) were obtained from Dharmacon (GE). Mena^INV^- and Mena11a-targeting siRNAs were purchased from Ambion (Custom Select siRNA). Transfection of siRNA and DNA into MDA-MB-231 cells was performed using the Lonza Nucleofector V kit, suspending 2 μM of siRNA or 1 μg of DNA in the included Lonza solution and electroporating 1 × 10^6^ cells using the Amaxa Nucleofector II device 24 h prior to the experiment for DNA constructs, and 72 h prior to the experiment for siRNA. Transfection of siRNA into MTLn3 cells was performed with Oligofectamine reagent (Life Technologies) 48 h prior the experiment, with an RNA concentration of 200 nM, except pan-Mena siRNA was used at 20 nM. DNA constructs were transfected at 1 ug/mL using Lipofectamine reagent (Life Technologies) as described previously^17^ 24 h prior to the experiment.

### Invadopodium staining and matrix degradation assay

A 0.2% gelatin (Sigma-Aldrich) solution was labeled with Alexa Fluor 405 dye (Molecular Probes, Eugene, OR) and MatTek dishes (MatTek Corporation, Ashland, MA) were coated with this conjugated gelatin as described elsewhere^87^. Briefly dishes were treated in 1N HCL before coating them with 50 μg/mL Poly-L-lysine (Sigma-Aldrich) and the Alexa 405 conjugated 0.2% gelatin diluted 1:40 in unlabeled 0.2% gelatin. This matrix was then crosslinked with a 0.1% glutaraldehyde solution, and the crosslinking quenched with 5 mg/ml sodium borohydride. Immediately prior to cell plating, MatTek dishes were pre-treated with media containing serum for 30 minutes, which we have found sufficient to add detectible levels of fibronectin. In cortactin phospho-mutant experiments, the invadopodium assay was performed on glutaraldehyde cross-linked, 0.2% gelatin coated dishes as described above, layered with Alexa Fluor 405-labeled fibronectin. 2 × 10^5^ MDA-MB-231 cells were plated on the Alexa Fluor 405-labeled matrices for 4 h, or 1 × 10^5^ MTLn3 cells for 12 h, at steady state prior to fixation in 4% PFA. Cells were permeabilized in 0.1% Triton X-100, blocked in a 2% BSA and 2% FBS solution in PBS, and primary and secondary antibodies were diluted in this same blocking solution. In experiments involving MDA-MB-231 cells stably expressing TagRFP-Cortactin-WT and –Cortactin-421F, endogenous human cortactin was knocked down 72 h prior to the start of the experiment and confirmed by immunoblot. As described elsewhere^62^, invadopodium precursors were defined as cortactin- and Tks5-rich puncta not co-localizing with a degradation hole in the Alexa Fluor 405-labeled gelatin, while those double-staining puncta that did co-localize with degradation holes were defined as mature invadopodia. For this assay, images of the fixed cells in PBS were taken on a DeltaVision Core Microscope (Life Sciences, GE) using a CoolSNAP HQ2 camera (Photometrics, Tuscon, AZ), a 60x oil objective with 1.4 numerical aperture (NA), a standard four-channel filter set and softWoRx software.

### Time lapse imaging of live cells for arrival kinetics

MTLn3 cells expressing eGFP-Mena, eGFP-Mena^INV^ or eGFP-Mena11a were transfected for 24 h with TagRFP-cortactin. 1 × 10^5^ cells were then plated on unlabeled gelatin overnight in complete media, serum-starved for 3-4 h in L15 media, and stimulated with 5 nM EGF to induce invadopodium precursor formation during imaging. The cells were imaged with frames taken every three seconds on a custom-built total internal reflection fluorescence (TIRF) microscope, used to minimize background, and the CRISP autofocus system (ASI, Eugene, OR) to stabilize the focus while images were collected using an Andor iXON^EM+^ EMCCD camera (Oxford Instruments), a 60x oil objective lens with 1.45 NA and a 2488-561-633rpc multi-bandpass mirror to enable multi-channel imaging.

### Phospho-cortactin assay and quantitative Immunofluorescence

3 × 10^5^ MDA-MB-231 cells were plated on unlabeled 0.2% gelatin matrix for 4 h prior to starvation in DMEM. Human EGF in L15 media at concentrations of 0.25 nM and 2.5 nM, or the L15-only control, was added for 3 minutes in a 37 °C water bath, and the cells were fixed in 4% PFA. PTP1B inhibition was accomplished by incubating cells in 10 μM PTP1B inhibitor dissolved in DMSO or an equal volume of DMSO alone, and diluted in L15 media, for 1 h prior to EGF treatment. PTP1B or control siRNA was transfected into cells at a concentration of 2 μM 48 h prior to EGF stimulation. pEGFP-PTP1B-HA C3 or pEGFP C3 plasmids were transfected into cells 24 h prior to EGF stimulation.

The intensity of cortactin and tyrosine 421 cortactin phosphorylation staining was quantified and expressed as the relative fold change in pY421-cortactin/cortactin ratio. Images for the invadopodium assay and tyrosine 421 cortactin phosphorylation staining were acquired on the DeltaVision Core Microscope (Applied Precision) with a CoolSNAP HQ2 camera, a 60x NA 1.4 oil objective, standard 4-channel filter set, and softWoRx image acquisition software.

### Statistical Analysis

Statistical analysis was performed with one-way analysis of variance (one way-ANOVA) analysis and Tukey’s multiple comparisons test or unpaired t-tests with Welch’s correction for unequal variance, using GraphPad Prism software. All bar graphs depict mean ± SEM, scatter plots depict 95% confidence intervals (CI).

## Additional Information

**How to cite this article**: Weidmann, M. D. *et al.* Mena^INV^ dysregulates cortactin phosphorylation to promote invadopodium maturation. *Sci. Rep.* **6**, 36142; doi: 10.1038/srep36142 (2016).

**Publisher’s note:** Springer Nature remains neutral with regard to jurisdictional claims in published maps and institutional affiliations.

## Supplementary Material

Supplementary Information
