# Supplementary material for: MenaINV dysregulates cortactin phosphorylation to promote invadopodium maturation
Source: Sci Rep. 2016 Nov 8;6:36142. doi: 10.1038/srep36142 (PMC5099927; doi:10.1038/srep36142)
Supplement: Supplementary Information [file srep36142-s1.pdf]

Mena[INV] dysregulates cortactin phosphorylation to promote invadopodium maturation.

Maxwell D. Weidmann[1*], Chinmay R. Surve[1,3], Robert J. Eddy[1], Xiaoming Chen[1], Frank B. Gertler[4], Ved P. Sharma[1,2] and John S. Condeelis[1,2,3*]

[1]Department of Anatomy and Structural Biology, [2]Gruss Lipper Biophotonics Center, USA, [3]Integrated Imaging Program, Albert Einstein College of Medicine, [4]Department of Biology and Koch Institute for Integrative Cancer Research, Massachusetts Institute of Technology.

*Corresponding authors

*Correspondence to maxwell.weidmann@med.einstein.yu.edu and john.condeelis@einstein.yu.edu

Supplemental Figure S1

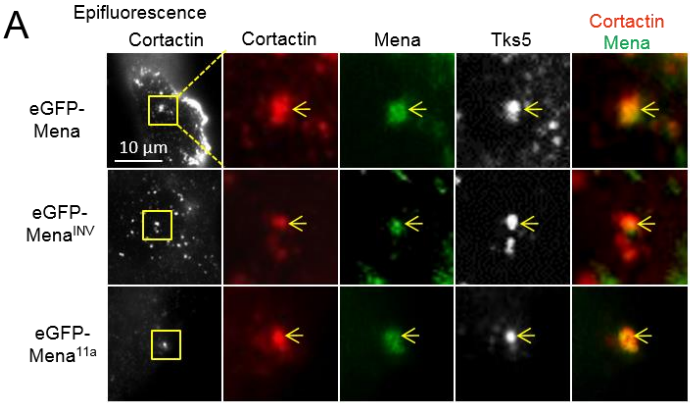

Supplementary Figure S1: Mena^{INV} localizes to invadopodia in breast carcinoma cell lines. A) Mena localizes to invadopodia. Representative images of MDA-MB-231 cells expressing eGFP-Mena, eGFP-Mena^{INV} or eGFP-Mena11a, plated on 0.2% gelatin, fixed and immunostained for Tks5 and cortactin. Yellow arrows indicate regions of co-localization in all 3 channels depicted.

Supplemental Figure S2

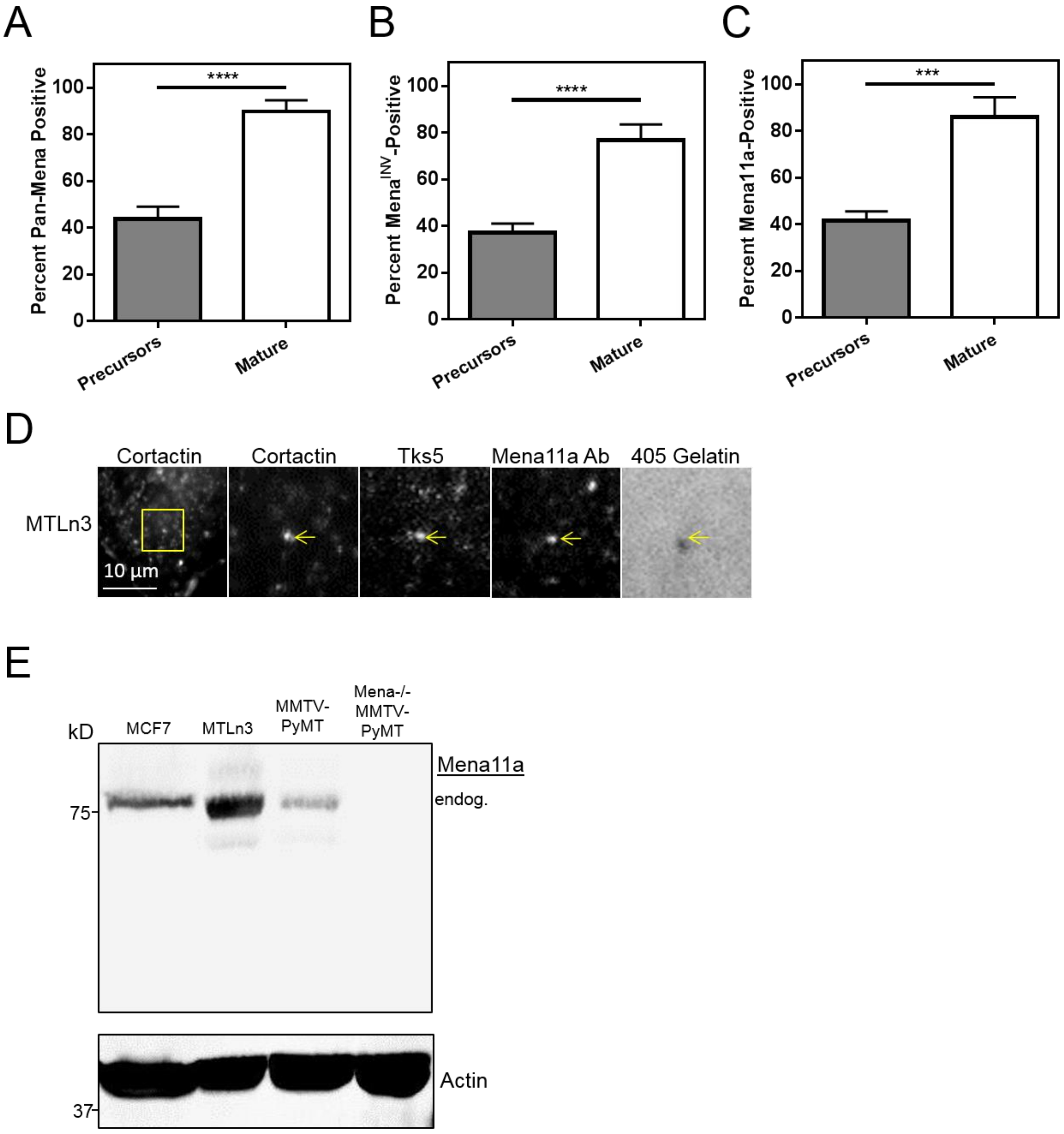

Supplementary Figure S2: Exogenously expressed Mena isoforms localize to mature invadopodia and to a lesser extent to invadopodium precursors. A-C) Mena isoforms preferentially localize at mature invadopodia compared to invadopodium precursors. The percentage of **A)** endogenous Mena isoforms (Pan-Mena), **B)** eGFP-Mena^{INV} and **C)** eGFP-Mena11a localizing to invadopodium precursors or mature invadopodia from the same images

shown in Fig. 1 (n > 90 invadopodia; 3 independent experiments). **D)** Mena11a localizes to mature invadopodia in MTLn3 cells. MTLn3 cells were plated on Alexa Fluor 405 labeled gelatin in complete media and immunostained for Tks5, cortactin, and an antibody specific for the 11a exon of Mena. **E)** Mena11a-specific antibody validation. Western blot of lysates from MCF7 cells, MTLn3 cells, tumor cells collected from mice bearing wild-type spontaneous MTTV-PyMT tumors, or Mena-null mice bearing spontaneous MTTV-PyMT tumors. Blotted lysates were stained with an antibody raised to the 11a exon of Mena, with β-actin used as a loading control. Data was analyzed for statistical significance by unpaired t-test with Welch's correction for unequal variance. *** $p < 0.001$, **** $p < 0.0001$.

Supplemental Figure S3

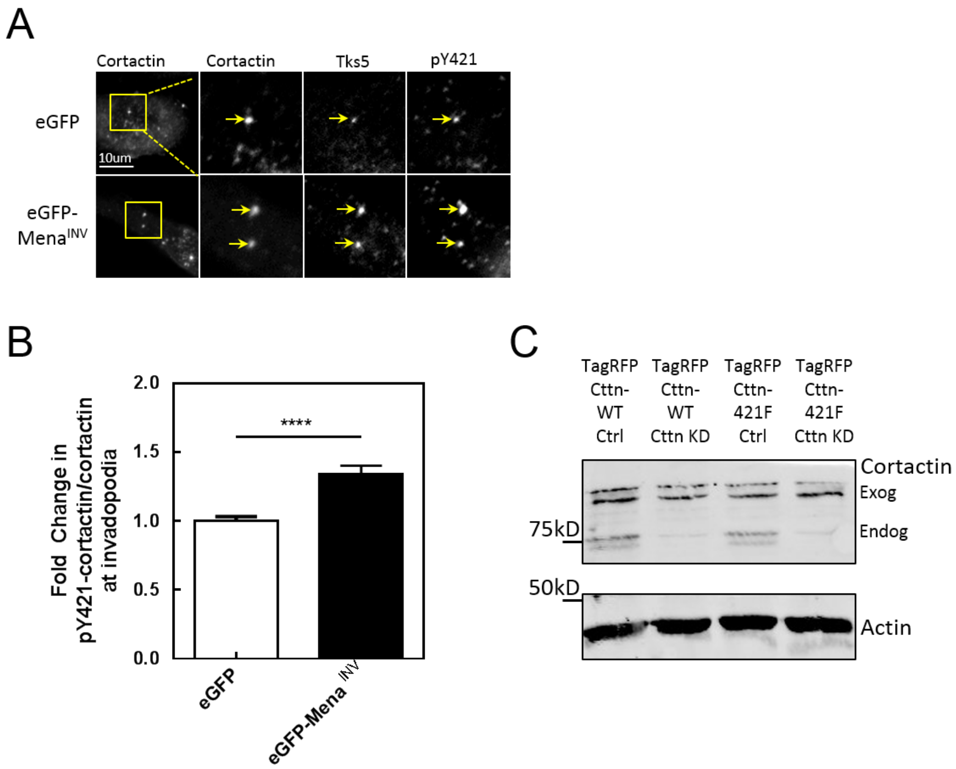

Supplementary Figure S**3**: **Mena^{INV} expression promotes cortactin phosphorylation at invadopodia in MTLn3 cells. A)** Mena^{INV} expression stimulates cortactin phosphorylation. Representative images of MTLn3 cells transduced with eGFP or eGFP-Mena^{INV} using a retrovirus that were plated on thin gelatin matrix and starved overnight. Cells were fixed and stained for cortactin, phospho-Y421 cortactin, and Tks5. **B)** Mena^{INV} expression stimulates cortactin phosphorylation. Quantification of cortactin phosphorylation expressed as the fold change in pY421-cortactin/cortactin ratio localized to punctate invadopodium precursors, defined as punctate co-localization of cortactin and Tks5, from images shown in A) (n > 100 invadopodia; three independent experiments). **C)** Endogenous cortactin knock down in MDA-MB-231 cells. Western blots of lysates from MDA-MB-231 cells used in Fig. 6, stably expressing either TagRFP-cortactin-WT or TagRFP-cortactin-Y421F, and treated with control siRNA or

siRNA targeting human cortactin. Blotted lysates were stained for cortactin and actin. Error bars indicate ±SEM, n > 100 invadopodia for each condition. Data was analyzed for statistical significance by unpaired t-test with Welch's correction for unequal variance. **** $p < 0.0001$.

Supplemental Figure S4

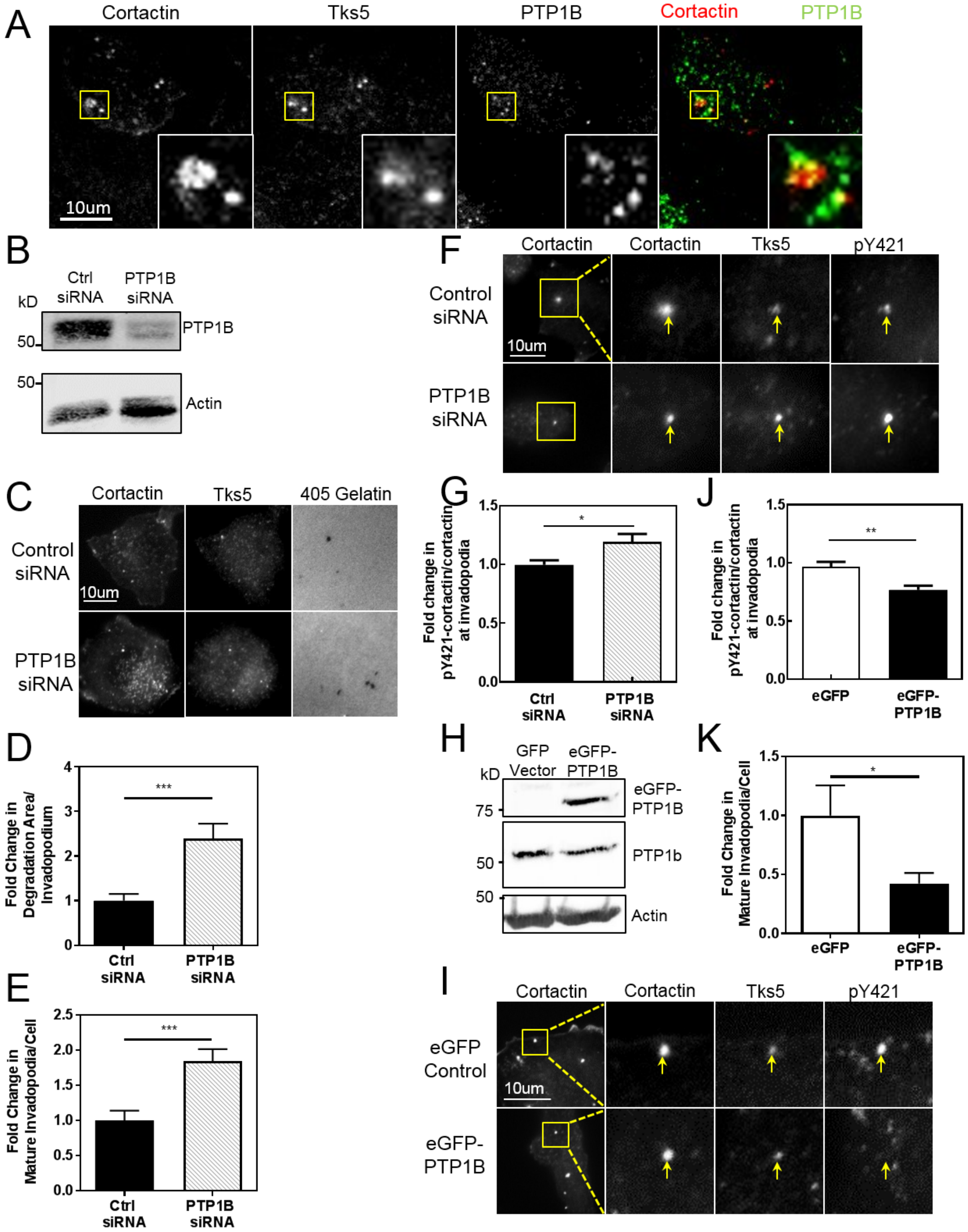

Supplementary Figure S4: **PTP1B localizes to invadopodia, where it suppresses cortactin phosphorylation in MTLn3 cells. A)** PTP1B localizes to invadopodia in MTLn3 cells. MTLn3 cells stained for cortactin, Tks5 and PTP1B, imaged on a TIRF microscope show PTP1B co-

localizing with cortactin- and Tks5-rich puncta. **B)** PTP1B KD in MTLn3 cells. Western blot of MTLn3 cells transfected with control or PTP1B single siRNA for 48 h and stained with a monoclonal antibody against PTP1B (EP1841Y), using β-actin as a loading control (n = three independent experiments). **C)** PTP1B KD increases invadopodium maturation in MTLn3 cells. Representative images of MLTn3 cells transfected with control or PTP1B siRNA for 48 h, plated on Alexa Fluor 405-labeled gelatin in complete media and immunostained for Tks5 and cortactin (n = 4 independent experiments). **D)** The average area of degraded Alexa Fluor 405-labeled gelatin under each MTLn3 cell was quantified from the images shown in C), normalized to the number of mature invadopodia per cell, and expressed as the fold-change relative to control siRNA-treated cells. **E)** The number of mature invadopodia per cell was quantified in the images shown in C), and expressed as the fold-change relative to control. **F)** PTP1B KD stimulates cortactin phosphorylation in MTLn3 cells. Representative images of MTLn3 cells transfected with control or PTP1B siRNA for 48 h, plated on thin gelatin matrix and starved overnight. Cells were then fixed and stained for cortactin, phospho-Y421 cortactin, and Tks5. **G)** Quantification of cortactin phosphorylation expressed as the fold change in pY421-cortactin/cortactin ratio localized to punctate invadopodium precursors, defined as punctate co-localization of cortactin and Tks5, from images shown in E) (n > 100 invadopodia; three independent experiments). **H)** eGFP-PTP1B overexpression in MTLn3 cells. Western blot of MTLn3 cells transfected with 1 µg of an empty pEGFP C3 vector or pEGFP-PTP1B-HA C3 DNA for 24 h and stained with monoclonal antibodies against PTP1B (EP1841Y) and GFP, using β-actin as a loading control (n = three independent experiments). **I)** eGFP-PTP1B overexpression suppresses cortactin phosphorylation in MTLn3 cells. Representative images of MTLn3 cells transfected as described in H) 24 h prior to fixation. Cells were then stained as in F). **J)** Images of MTLn3 cells described in I) were then quantified as in G). (n > 100 invadopodia; three independent experiments). **K)** eGFP-PTP1B overexpression suppresses invadopodium maturation in MTLn3 cells. MTLn3 cells treated as in H) were stained as in C) and the number of mature invadopodia per cell

quantified ($n > 60$ cells; three experiments). Error bars indicate ±SEM. Data was analyzed for statistical significance by unpaired t-test with Welch's correction for unequal variance. * $p < 0.05$, ** $p < 0.01$, *** $p < 0.001$.
